# Planned vaginal and planned cesarean delivery outcomes in pregnancies complicated with pregestational type 1 diabetes – A three-year academic tertiary hospital cohort study

**DOI:** 10.1186/s12884-022-04510-8

**Published:** 2022-03-02

**Authors:** Heidi Kruit, Saara Mertsalmi, Leena Rahkonen

**Affiliations:** 1 Department of Obstetrics and Gynecology, University of Helsinki and Helsinki University Hospital, Haartmaninkatu 2, 00029 HUS, Helsinki, Finland; 2 Department of Internal Medicine, Hyvinkää Hospital Sairaalankatu 1, 05850 Hyvinkää, Finland

**Keywords:** Diabetes mellitus type 1, Pregestational diabetes, Adverse maternal outcome, Adverse perinatal outcome, Unscheduled caesarean delivery, Induction of labor

## Abstract

**Background:**

Finland has the world’s highest incidence of 62.5/100000 of diabetes mellitus type 1 (DM1) with approximately 400 (1%) DM1 pregnancies annually. Pregnancies complicated by DM1 are accompanied with increased risk for perinatal morbidity and mortality. Timing and mode of delivery are based on the risk of complications, yet the data on labor induction is limited. The aim of this study was to compare delivery outcomes in planned vaginal (VD) and planned cesarean deliveries (CD) in late preterm and term DM1 pregnancies, and to evaluate the feasibility of labor induction.

**Materials and Methods:**

Pregnant women with DM1, live singleton fetus in cephalic presentation ≥34 gestational weeks delivering in Helsinki University Hospital between January 1st 2017 and December 31st 2019 were included. The primary outcome were the rates of adverse maternal and perinatal outcome. The study population was classified according to the 1980-revised White’s classification. Statistical analyses were performed by IBM SPSS Statistics for Windows.

**Results:**

Two hundred four women were included, 59.8% (*n* = 122) had planned VD. The rate of adverse maternal outcome was 27.5% (*n* = 56), similar between the planned modes of delivery and White classes. The rate of perinatal adverse outcome was 38.7% (*n* = 79), higher in planned CD (52.4% vs. 29.5%;*p* = 0.001). The most common adverse perinatal event was respiratory distress (48.8% vs. 23.0%;*p* <  0.001). The rate of adverse perinatal outcome was higher in White class D + Vascular compared to B + C (45.0% vs. 25.0%, OR after adjustment by gestational age 2.34 [95% CI 1.20–4.50];*p* = 0.01). The total rate of CD was 63.7% (*n* = 130), and 39.3% (*n* = 48) in planned VD. Women with White class D + Vascular more often had emergency CD compared to White Class B + C (48.6% vs. 25.0%;*p* = 0.009). The rate of labor induction was 51%, being 85.2% in planned VD. The rate of VD in induced labor was 58.7% (*n* = 61) and the rate of failed induction was 14.1% (*n* = 15).

**Conclusion:**

Planned VD was associated with lower rate of adverse perinatal outcome compared to planned CS, with no difference in the rates of adverse maternal outcome. Induction of labor may be feasible option but should be carefully considered in this high-risk population.

## Background

Finland has the world’s highest incidence of 62.5/100000 of diabetes mellitus type 1 (DM1), an autoimmune disease leading to insulin therapy and development of vascular, renal, and neuropathic complications [1, 2]. Approximately 400 (1%) pregnancies are complicated by DM1 annually [3]. The perinatal mortality rate has decreased over the decades, but pregnancies complicated by pregestational diabetes are still accompanied with increased risk for perinatal morbidity and mortality [4, 5]. Risk for adverse perinatal outcome is the lowest with optimal glucose control achieved prior to pregnancy, and in the absence of maternal vascular complications, emphasizing the importance of pre-pregnancy counselling and multidisciplinary follow-up [6, 7].

The classification of maternal diabetes, based on the age at onset and duration of diabetes and presence of complications, and their relationship to adverse pregnancy outcome, was first introduced by Priscilla White in 1949 [8]. In the 1980 revision of White’s classification, the presence of chronic hypertension reclassified the women with classes B and C as class D [9].

Optimal timing and delivery are based on glycemic control, fetal growth, and pregnancy complications. In Helsinki University Hospital, in which majority of the DM1 pregnancies of Finland are treated, delivery by 37–38 gestational weeks is usually indicated [6]. In women with poor glycemic control, diabetic complications, or macrosomic fetus, delivery is planned earlier [6]. However, only limited data exist on induction of labor in women with pregestational DM1.

The aim of this study was to compare delivery outcomes in planned vaginal delivery (VD) and planned caesarean delivery (CD) in late preterm and term pregnancies complicated by pregestational DM1, and to evaluate the feasibility of labor induction in these women.

## Materials and Methods

This retrospective cohort study included all pregnant women with pregestational DM1, live singleton pregnancies in cephalic presentation ≥34 gestational weeks in Helsinki University Hospital between January 1st 2017 and December 31st 2019. The study protocol was approved by the institutional review board of the hospital region (Helsinki and Uusimaa Hospital District Committee for Obstetrics and Gynecology, HUS/3172/2018 and HUS/54/2019). Due to the retrospective nature of the study, written informed consent was waived according to national legislation (Medical Research Act 488/1999, chapter 2 a (23.4.2004/295), section 5 and 10a).

The primary outcome were the rates of adverse maternal and perinatal outcome. Maternal adverse outcome was defined as maternal death, admission to intensive care unit, uterine rupture, hysterectomy, post-partum hemorrhage ≥1000 ml, preeclampsia,, and intrapartum infection or post-partum infection. Perinatal adverse outcome was defined as stillbirth, neonatal death, shoulder dystocia, birth injury defined as brachial plexus injury or fracture, neonatal seizures, umbilical artery blood pH ≤7.05 at birth, umbilical artery blood base excess ≤ − 12, 5-min Apgar-score < 7, and neonatal infection.

The data on baseline characteristics and delivery outcomes were collected in the hospital database. Maternal baseline characteristics included age, height, pre-pregnancy body mass index (BMI), smoking, age of DM1 onset, diabetes complications, previous pregnancies and deliveries. The collected delivery parameters included the induction to delivery interval, mode of delivery, indication for cesarean section (CS), post-partum hemorrhage, perineal tear, placental retention, intrapartum and postpartum infections, birthweight, Apgar score, umbilical artery blood gas values, admission to maternal or neonatal intensive care unit, and maternal birth experience.

All women with pregestational diabetes were routinely scheduled for prenatal visits every 4 weeks until 28 gestational weeks, and every 2 weeks from 28 weeks until delivery. Prenatal monitoring included ultrasound assessment of fetal growth and wellbeing, maternal blood pressure and urine sampling, and multidisciplinary assessment of glucose monitoring and diabetes treatment. Continuous glucose monitoring measuring the glucose content of interstitial fluid through a needle sensor inserted subcutaneously and short- or rapid-acting insulin analogues were routinely used during pregnancy. The patient shared their glucose scanning data with the maternity clinic via an online application. Hemoglobin A1c (HbA_1c_) was routinely measured every 4 weeks, and diabetic eye screening was performed in the early pregnancy.

The study population was classified according to the 1980-revised White’s classification [9]. Women with pregestational hypertension were upstaged to class D [10]. Nephropathy was defined as ≥500 mg/dl proteinuria in the early pregnancy. Classes F, R, RF, and H were combined to create a “Vascular class” due to the small number of patients representing each of these groups. Proliferative retinopathy, nephropathy or cardiovascular heart disease were considered a vascular complication. Preeclampsia was diagnosed as blood pressure readings ≥140/90 mmHg with either proteinuria ≥0.5 g/day or evidence of end-organ involvement including symptoms and abnormal preeclampsia laboratory values. Fetal macrosomia was defined as a combination of either growth above 95th percentile for the given gestational age (3500 g at 36 weeks of gestation, 3700 g at 37 weeks, and 3900 g at 38 weeks) or the absolute birthweight of ≥4500 g [11].

Induction of labor was carried out by oral 50 mcg misoprostol administered every 4 h or a single 40–80 ml balloon catheter (Rüsch 2-way Foley Couvelaire tip catheter size 22 Ch, Teleflex Medical, Athlone, Ireland) retained for a maximum of 24 h. When Bishop score ≥ 6 was reached, labor induction was continued by amniotomy in case of intact amniotic membranes, and oxytocin in the absence of spontaneous contractions. Continuous intrapartum electronic fetal monitoring and glucose monitoring minimally every hour during labor was routinely used. Failed induction was diagnosed after ruptured membranes and 12 h of oxytocin administration without cervical change [12].

Shoulder dystocia was defined as delivery that required additional obstetric maneuvers to deliver the fetus after the head was delivered and gentle traction had failed. Maternal satisfaction in birth experience was measured after delivery using a Visual Analog Scale (VAS) score [13], where zero stands for the most negative experience possible and ten for the most positive experience possible. VAS-score < 5 was defined as low birth experience score.

The data are expressed as n (%) and means (SD) and median (range) as separately indicated. Statistical analyses were performed by using IBM SPSS Statistics for Windows, Version 26.0 (Armonk, NY, USA). Categorical variables were analyzed for odds ratios (OR) with 95% confidence interval (CI). Categorical variables were compared by the chi-square test and Fisher’s exact test when appropriate. Data with continuous variables were performed by T-test when the data followed normal distribution and by a Mann-Whitney U test if the data did not follow normal distribution. Unadjusted and adjusted odd ratios (OR) with 95% confidence intervals (CI) were calculated for the primary outcome by modelling the data to control for gestational age. A *p*-value < 0.05 was considered statistically significant.

## Results

The study population included 204 women with pregestational DM1. The planned mode of delivery was planned CD in 40.2% (*n* = 82) and planned VD in 59.8% (*n* = 122) of the women. The mean age of then women was 31.1 (SD 5.0) years, the mean BMI 25.3 (SD 4.5), and the mean gestational age 37.1 (SD 1.1) weeks. The median age of receiving DM1 diagnosis was 12.0 (range 1–37) years. The characteristics and the modified White classification [11] of the study population are presented in Table 1. One or more diabetic complication prior to the pregnancy were present in 56.9% of the study population.

**Table 1:** Characteristics and the modified White classification [10] of the study population *N* = 204

	Planned vaginal delivery		Planned caesarean delivery		***p***-value
	***n*** = 122	%	***n*** = 82	%	
Nulliparous	70	57.4	32	39,0	0.01
Maternal age ≥ 35	26	21.3	24	29.3	0.20
Maternal height < 164 cm	22	26.8	36	29.5	0.68
Pre-pregnancy BMI ≥30	16	13.1	16	19.5	0.22
Previous caesarean section	17	13.9	46	56.1	< 0.001
Previous vaginal delivery	38	31.1	5	6.1	< 0.001
Smoking	13	10.7	7	8.5	0.62
IVF	4	3.3	3	3.7	0.88
Induction of labor	104	85.2	NA		
Onset age of DM1					
< 10 years	42	34.4	33	40.2	0.40
10–19 years	49	40.2	35	42.7	0.72
≥ 20 years	31	25.4	14	17.1	0.16
Duration of DM1					
< 10 years	34	27.9	11	13.4	0.02
10–19 years	38	31.1	28	34.1	0.65
≥ 20 years	50	41.0	43	52.4	0.11
Diabetes complications					
Retinopathy	54	44.3	55	67.1	0.001
Proliferative retinopathy	2	1.6	5	6.1	0.12
Nephropathy	4	3.3	3	3.7	0.88
Neuropathy	1	0.8	5	6.1	0.04
Pregestational hypertension	3	2.5	4	4.9	0.35
Preeclampsia	8	6.6	7	8.5	0.60
The Modified White Class					
Class B^a^	22	18.0	7	8.5	0.06
Class C^b^	26	21.3	9	11.0	0.06
Class D^c^	68	55.7	60	73.2	0.01
Class R^d^	2	1.6	3	3.7	0.36
Class F^e^	4	3.3	1	1.2	0.35
Class RF^f^	0	0	2	2.4	0.16
Class “Vascular complication” ^g^	6	4,9	6	7,3	0.45

^a^Class B; Onset age ≥ 20 years or duration < 10 years, no complications

^b^Class C; Onset age 10–19 years or duration 10–19 years, no complications

^c^Class D; Onset age of < 10 years or duration ≥20 years, background retinopathy or hypertension

^d^Class R; Proliferative retinopathy or vitreous haemorrhage

^e^Class F; Nephropathy with over 500 mg/dl proteinuria

^f^Class RF; Criteria for both Classes R and F coexist

^g^Class “Vascular”; Classes F, R, RF, and H were combined to create a “Vascular” class

The delivery outcomes are shown in Table 2. The total rate of adverse maternal outcome composite was 27.5% (*n* = 56), being similar in planned VD and planned CD (Table 2). No cases of maternal death, admission to intensive care unit, uterine rupture, or hysterectomy occurred. The most common maternal adverse event was post-partum hemorrhage ≥1000 ml, which occurred in 17.2% (*n* = 35) of the women, with no difference between the planned modes of delivery (Table 2).

**Table 2:** Delivery outcomes in planned vaginal and planned caesarean delivery N = 204

	Planned vaginal delivery		Planned caesarean delivery		***p***-value
	***n*** = 122	%	***n*** = 82	%	
Vaginal delivery	74	60.7	0		
Operative vaginal delivery	15	12.3	0		
**Emergency caesarean section**	48	39.3	5	6.1	< 0.001
Fetal distress	24	50.0	0		
Labor arrest	23	47.9	0		
Preeclampsia	1	2.1	5	6.1	
**Induction of labor**	104	85.2	NA		
Vaginal delivery in induced labor	61	58.7	NA		
Failed induction	15	14.4	NA		
Preterm delivery (≤ 36 + 6 weeks)	78	63.9	41	50.0	0.05
Birthweight [mean (SD)]	3668	444	3965	583	< 0.001
Fetal macrosomia^a^	36	29.5	46	56.1	< 0.001
Birthweight ≥4500 g	2	1.6	15	18.3	< 0.001
**Number of women with composite adverse maternal outcome**^**b**^	32	26.2	24	29.3	0.63
**Number of instances of maternal morbidity**	38	31.15	25	30.5	0.66
Maternal death	0		0		
Admission to intensive care	0		0		
Uterine rupture	0		0		
Hysterectomy	0		0		
Post-partum hemorrhage ≥1000 ml	21	17.2	14	17.1	0.98
Preeclampsia	8	6.6	7	8.5	
Maternal infection	9	7.4	4	4.9	0.47
Intrapartum infection	4	3.3	0	0.0	0.10
Post-partum infection	5	4.1	4	4.9	0.79
Sphincter injury	1	0.8	NA		
Maternal birth experience on visual analog scale (VAS) score < 5	15	13.2	4	5.1	0.07
**Number of neonates with composite adverse perinatal outcome**^**c**^	36	29.5	43	52.4	0.001
**Number of events of perinatal morbidity**	58	47.5	50	61.0	< 0.001
Stillbirth	0		0		
Neonatal death	0		0		
Shoulder dystocia	4	3.3	0		
Birth injury	3	2.5	0		
Respiratory distress	28	23.0	40	48.8	0.58
Neonatal seizures	1	0.8	0		
Umbilical artery blood pH ≤ 7.05	5	4.1	1	1.2	0.23
Umbilical artery blood BE ≤12	3	2.5	0		
5-min Apgar-score 7 or less	14	11.5	9	11.0	0.97
Neonatal infection	0	0.0	0	0.0	
**Neonatal intensive care unit admission**	63	51.6	56	68.3	0.02
Respiratory distress	28	23.0	40	48.8	< 0.001
Hypoglycemia	12	9.8	5	6.1	0.36
Prematurity	21	17.2	10	12.2	0.38
Asphyxia	2	1.6			
Heart anomaly	0		1	1.2	

^a^ Fetal macrosomia defined as growth above 90th percentile for the given gestational age or the absolute birthweight of ≥4500 g

^b^Adverse maternal outcome composite included maternal death, admission to intensive care unit, uterine rupture, hysterectomy, post-partum hemorrhage ≥1000 ml, preeclampsia, shoulder dystocia, and maternal intrapartum or post-partum infection

^c^Adverse perinatal outcome composite included stillbirth, neonatal death, birth injury, respiratory distress, neonatal seizures, umbilical artery blood pH ≤ 7.05 at birth, umbilical artery blood base excess (BE) ≤ 12, 5-min Apgar-score 7 or less, and neonatal infection

The rate of adverse perinatal outcome composite in the study population was 38.7% (*n* = 79), being higher in planned CD compared to planned VD (29.5% vs. 52.4%; *p* = 0.001, OR 2.6 [95% CI 1.5–4.7]) (Table 2). No cases of stillbirth or neonatal death occurred. The most common perinatal adverse event (33.3%, *n* = 68) was respiratory distress requiring ventilatory treatment, being higher in planned CS (23.0% in planned VD vs. 48.8% in planned CD; *p* <  0.01). Neonatal intensive care unit (NICU) admission was more common in the planned CD compared to planned VD (68.3% vs. 51.6%; *p* = 0.02). The most common reason for NICU admission was respiratory distress in both groups (Table 2). The total rate of fetal macrosomia was 40.2%, being higher in planned CD (Table 2). The rate of shoulder dystocia in VD was 5.4% (*n* = 4/74). Two of the cases of shoulder dystocia occurred in White Class B women, one in White class D, and one in Vascular Class. Three of the women underwent induction of labor, and one woman in White Class B had spontaneous onset of labor. The respective birthweight and gestational age were 4230 g (35 + 2), 3440 g (38 + 0), 3940 g (37 + 2) and 3700 g (38 + 1).

The total rate of caesarean delivery in the cohort was 63.7% (*n* = 130). Of the women with planned VD, 39.3% (*n* = 48) had unscheduled CS. The women with one or more diabetic complication less frequently achieved VD compared to the women with no diabetic complication [50.0% (*n* = 28) vs 69.7% (*n* = 46); *p* = 0.026]. The main indications for unscheduled CS were fetal distress in 50.0% and labor arrest in 47.9% of the cases (Table 2). The unscheduled CS and VD rates of the women with planned VD according to the modified White classification are presented in Table 3. The rate of unscheduled CS was the highest in White Class D (Table 3). The rates of composite adverse maternal outcome did not differ between the White Classes (Table 3) in planned VD (White class B + C 25.0% vs. White class D + Vascular 27.0%; *p* = 0.80, and after adjustment by gestational age OR 1.06 [95% CI 0.46–2.45]; *p* = 0.90). The rate of adverse perinatal outcome composite was higher in White class B compared to White class C, and in White class D and Vascular combined to combined White class B and C (Table 3). After adjustment with gestational age, the risk for adverse perinatal outcome with planned VD in White class D and Vascular was similar to combined White class B and C (OR 1.699 [95% CI 0.74–3.92]; *p* = 0.21).

**Table 3:** The rates of emergency caesarean delivery, adverse maternal and adverse perinatal outcome in the women with planned vaginal delivery according to the modified White classification n = 122

	Class B (***n*** = 22)	Class C (***n*** = 26)	Class B and C (n = 48)	Class D and Vascular (***n*** = 74)	***p***- value^**a**^	***p***- value^**b**^	***p***- value^**c**^
	n (%)	n (%)	n (%)	n (%)			
Emergency caesarean delivery	7 (31.8)	5 (19.2)	12 (25.0)	36 (48.6)	0.32	0.009	0.009
Adverse maternal outcome	6 (27.3)	6 (23.1)	12 (25.0)	20 (27.0)	0.74	0.69	0.80
Adverse perinatal outcome	8 (36.4)	3 (11.5)	11 (22.9)	25 (33.8)	0.04	0.03	0.20

^a^*p*- value comparing the difference between Class B and Class C

^b^*p*- value comparing the difference between Class C and Class D

^c^*p*- value comparing the difference between Classes B + C and Class D + Vascular

Table 4 presents the rates of adverse maternal and perinatal outcome composites according to the modified White classification and the planned mode of delivery. The rates of adverse maternal outcome did not differ between the planned delivery modes and the White classes (White class D and Vascular compared classes B and C combined 30.0% vs. 21.9%, OR 1.53 [95% CI 0.77–2.06], and after adjustment by gestational age OR 1.44 [95% CI 0.7–2.9]; *p* = 0.31) (Table 5). The rate of adverse perinatal outcome was higher in White class D and Vascular compared to classes B and C combined (45.0% vs. 25.0%, unadjusted OR 2.46 [95% CI 1.27–4.73], and after adjustment by gestational age OR 2.34 [95% CI 1.2–4.5]; *p* = 0.01) (Table 5).

**Table 4:** The rates of adverse delivery outcome according to the planned mode of delivery in the modified White classes = 204

	Class B (***n*** = 29)			Class C (n = 35)			Class D (***n*** = 128)			Class D and Vascular (***n*** = 140)		
	Planned vaginal delivery n = 22 (%)	Planned cesarean delivery n = 7 (%)	***p***- value	Planned vaginal delivery n = 26 (%)	Planned cesarean delivery ***n*** = 9 (%)	***p***-value	Planned vaginal delivery n = 68 (%)	Planned cesarean delivery ***n*** = 60 (%)	***p***-value	Planned vaginal delivery n = 74 (%)	Planned cesarean delivery ***n*** = 66 (%)	***p***-value
**Adverse maternal outcome**	6 (27.3)	1 (14.3)	0.65	6 (23.1)	1 (11.1)	0.65	19 (27.9)	20 (33.3)	0.51	20 (27.0)	22 (33.3)	0.46
**Adverse perinatal outcome**	8 (36.4)	2 (28.6)	1.0	3 (11.5)	3 (33.3)	0.16	22 (32.4)	34 (56.7)	0.006	25 (33.8)	38 (57.6)	0.006

**Table 5:** The unadjusted and adjusted odds ratios with 95% confidence intervals for composite of adverse maternal and adverse perinatal outcome in White Classa B and C compared to White Class D and vascular women

	Class B and C (***n*** = 64) n (%)	Class D and Vascular (n = 140) n (%)	Unadjusted OR (95% CI)	Adjusted^a^ OR (95% CI)	***p***-value
**Adverse maternal outcome**	14 (21.9)	42 (30.0)	1.53 (0.77–2.06)	1.44 (0.7–2.90)	0.31
**Adverse perinatal outcome**	16 (25.0)	63 (45.0)	2.46 (1.27–4.73)	2.34 (1.20–4.50)	0.01

^a^ Adjusted by gestatiol age

The rate of labor induction was 51% (*n* = 104), being 85.2% (n = 104/122) in the women with planned vaginal delivery. The median induction to delivery interval was 31.4 (range 1.9–104.2) h. The rate of vaginal delivery in induced labor was 58.7% (*n* = 61), and the rate of failed induction was 14.1% (*n* = 15). Induction of labor was more often successful in White class B + C compared to White D + Vascular class (73.2% [*n* = 30/41] vs. 49.2% [*n* = 31/63]; *p* = 0.02). The rates of adverse maternal outcome (76.0% [*n* = 79/104] vs. 83.3% [n = 15/18]; *p* = 0.49) and adverse perinatal outcome (13.5% [*n* = 14/104] vs. 27.8% [*n* = 5/18]; *p* = 0.12) were similar in women with induced and spontaneous onset of labor.

## Discussion

This study was a three-year tertiary referral hospital cohort assessing maternal and perinatal adverse outcomes according to the planned mode of delivery and the White classification in 204 late preterm or term pregnancies complicated by pregestational DM1. Our results suggest that planned VD is associated with lower rate of adverse perinatal outcome compared to planned CD in DM1 pregnancies, with no difference in the rates of adverse maternal outcome. The most common adverse perinatal outcome and reason for NICU admittance was respiratory distress requiring ventilatory treatment. The rate of unscheduled CS was higher, being almost 50%, in White Class D + Vascular women compared to White Class B + C women. Induction of labor resulted in vaginal delivery in approximately 60% of the women, with 14.1% induction failure, and may be a feasible option.

The strength of this study is the considerable size of the cohort, and the setting in a country of the highest incidence of DM1 in the world. The established management practices of our tertiary referral hospital with 14,000 deliveries annually, and the uniform guidelines of pregnancy and labor management in women with pregestational diabetes enable realistic comparisons of delivery outcomes. The weakness of the study is the retrospective design that biases the conclusions of the study, since the women with planned CD and the women with planned VD are inherently different. However, in this high-risk population with high rate of fetal macrosomia and risk for shoulder dystocia and mortality, a randomized trial on the mode of delivery would not be feasible. We also regret not having the data on the HbA_1c_ – values prior to and during the pregnancy or information on pre-pregnancy counselling.

In our study, almost 60% of the women had at least one diabetic complication, retinopathy being the most common. Retinopathy commonly progresses in pregnancy [14, 15], but this was not seen in our study. The occurrence of nephropathy and hypertension were lower in our study than the previously reported rates of 5–10% [15–17]. The rate of fetal macrosomia, associated with increased risk for shoulder dystocia, and perinatal mortality and morbidity was lower (40%) in our study compared to the previously reported estimates of 60% [18–20]. However, the definitions for macrosomia in the previous publications range from the absolute birthweight of 4000 or 4500 g to birthweight above the 90th percentile for the given gestational age [18–20]. In our study, both definitions were combined to include all cases of macrosomia [11].

Bennet’s validation of the 1980’s revised White classification showed the classification still helpful in providing information for assessing women with DM1 [10]. In Bennett’s study, the incidence of macrosomia and shoulder dystocia decreased as the class increased toward the vascular categories, whereas the incidence of small for gestational age fetus and preeclampsia increased [11]. Controversially, in our study the birthweight was greater in White Class D compared to White class C, but half of the shoulder dystocia cases occurred in White class B. The lower rates of diabetic complications in our study may partly be explained by the routine use of continuous glucose monitors and frequent HbA_1c_ monitoring every 4 weeks throughout the pregnancy, as better glucose control in the second and third trimester is associated with the lowest risk of fetal macrosomia and complications [7, 18, 21, 22]. Furthermore, the multidisciplinary approach for pregnancy monitoring used in our clinic, has also previously been reported to result in better maternal and neonatal outcomes [23].

The total rate of adverse maternal outcome in the study did not differ between the modes of delivery or the White classes. The rate of adverse perinatal outcome was higher in planned CD, with respiratory distress requiring ventilatory treatment being the most common adverse event. Respiratory distress was significantly more frequent in neonates delivered by planned CS compared to planned VD. The relatively high neonatal intensive care admission rate may partly be explained by the respiratory distress, the 60% rate of preterm birth, and the hospital policy of low threshold in admitting the neonates born to mothers with DM1 to intensive care unit for monitoring for hypoglycemia.

Limited data exists on labor induction in pregnancies complicated by pregestational DM1. In our cohort, almost nine of ten women aiming for vaginal delivery were induced. Labor induction resulted in successful vaginal delivery in approximately 60% of the women, being more often successful in White class B and C compared to White D and Vascular class. The rate of unscheduled CS was 40%, as also reported previously [24], and was the highest in White class D. Furthermore, the highest rates of adverse maternal and perinatal outcome occurred in White D + Vascular class diabetic women with failed attempt of VD and emergency cesarean delivery. Pregestational diabetes has been associated with increased rates of uterine rupture [25], but this was not seen in our study.

In conclusion, our results suggest that planned vaginal delivery, including induction of labor, is associated with lower rates of adverse perinatal outcome compared to planned cesarean delivery, with no difference in the rates of adverse maternal outcome. Induction of labor may a feasible option, although the decision on labor induction should be carefully considered in this high-risk population, especially in women with White class D and Vascular DM1.

## Data Availability

The datasets generated and analyzed during the current study are not publicly available due to institutional policy and limitations of ethical approval involving the patient data and anonymity but are available from the corresponding author on reasonable request.

## Abbreviations

BMI: Body mass index
CD: Cesarean delivery
CI: Confidence interval
CS: cesarean section
DM1: Diabetes mellitus type 1
OR: Odds ratio
VAS: Visual Analog Scale
VD: Vaginal delivery

## References

[1] Harjutsalo V, Sund R, Knip M (2013). Incidence of type 1 diabetes in Finland. JAMA..

[2] Soltesz G, Patterson CC, Dahlquist G, EURODIAB Study Group (2007). Worldwide childhood type 1 diabetes incidence--what can we learn from epidemiology?. Pediatr Diabetes.

[3] Vuori E, Gissler M, National institute of Finland for Health and Welfare (2019). Perinatal statistics: Parturients, deliveries and newborns 2018.

[4] Bell R, Bailey K, Cresswell T (2008). Trends in prevalence and outcomes of pregnancy in women with pre-existing type I and type II diabetes. BJOG..

[5] Feig DS, Hwee J, Shah BR, Booth GL, Bierman AS, Lipscombe LL (2014). Trends in incidence of diabetes in pregnancy and serious perinatal outcomes: A large, population-based study in ontario, canada, 1996-2010. Diabetes Care.

[6] Working group set up by the Finnish Medical Society Duodecim and the Finnish Cardiac Society (2013). Gestational diabetes. Current care guidelines.

[7] American Diabetes Association (2021). 14. management of diabetes in pregnancy: Standards of medical care in diabetes-2021. Diabetes Care.

[8] White P (1949). Pregnancy complicating diabetes. Am J Med.

[9] White P (1978). Classification of obstetric diabetes. Am J Obstet Gynecol.

[10] Bennett SN, Tita A, Owen J, Biggio JR, Harper LM (2015). Assessing white's classification of pregestational diabetes in a contemporary diabetic population. Obstet Gynecol.

[11] Boulvain M, Senat MV, Perrotin F, Winer N, Beucher G, Subtil D (2015). Induction of labour versus expectant management for large-for-date fetuses: a randomised controlled trial. Lancet..

[12] Caughey AB, Cahill AG, Guise JM, Rouse DJ, American College of Obstetricians and Gynecologists (College), Society for Maternal-Fetal Medicine (2014). Safe prevention of the primary caesarean delivery. Am J Obstet Gynecol.

[13] Pham CT, Crowther CA (2003). Birth outcomes: Utility values that postnatal women, midwives and medical staff express. BJOG..

[14] Egan AM, McVicker L, Heerey A, Carmody L, Harney F, Dunne FP (2015). Diabetic retinopathy in pregnancy: A population-based study of women with pregestational diabetes. J Diabetes Res.

[15] Rosenn B, Miodovnik M, Kranias G (1992). Progression of diabetic retinopathy in pregnancy: Association with hypertension in pregnancy. Am J Obstet Gynecol.

[16] Yanit KE, Snowden JM, Cheng YW, Caughey AB (2012). The impact of chronic hypertension and pregestational diabetes on pregnancy outcomes. Am J Obstet Gynecol.

[17] Combs CA, Rosenn B, Kitzmiller JL, Khoury JC, Wheeler BC, Miodovnik M (1993). Early-pregnancy proteinuria in diabetes related to preeclampsia. Obstet Gynecol.

[18] Cyganek K, Skupien J, Katra B (2017). Risk of macrosomia remains glucose-dependent in a cohort of women with pregestational type 1 diabetes and good glycaemic control. Endocrine..

[19] Kitzmiller JL, Block JM, Brown FM (2008). Managing preexisting diabetes for pregnancy: Summary of evidence and consensus recommendations for care. Diabetes Care.

[20] Macrosomia: ACOG practice bulletin, number 216. Obstet Gynecol. 2020;135(1):e18–35. 10.1097/AOG.0000000000003606. 10.1097/AOG.0000000000003606 31856124

[21] Voormolen DN, DeVries JH, Sanson RME (2018). Continuous glucose monitoring during diabetic pregnancy (GlucoMOMS): A multicentre randomized controlled trial. Diabetes Obes Metab.

[22] Feig DS, Donovan LE, Corcoy R (2017). Continuous glucose monitoring in pregnant women with type 1 diabetes (CONCEPTT): A multicentre international randomised controlled trial. Lancet..

[23] Yoeli-Ullman R, Dori-Dayan N, Mazaki-Tovi S (2020). Towards implementation of the saint vincent declaration: Outcomes of women with pregestational diabetes. Isr Med Assoc J.

[24] Dharan VB, Srinivas SK, Parry S, Ratcliffe SJ, Macones G (2010). Pregestational diabetes: A risk factor for vaginal birth after caesarean section failure?. Am J Perinatol.

[25] McLaren RAJ, Ndubizu C, Atallah F, Minkoff H (2020). Association of uterine rupture with pregestational diabetes in women undergoing trial of labour after caesarean delivery. J Matern-Fet Neonat Med.

